# Hypoxic Training with Calorie Restriction Improves Lipid Profile and Body Composition in Men with Obesity-Related Hypercholesterolemia: A Controlled Intervention Study

**DOI:** 10.3390/ijms262211048

**Published:** 2025-11-14

**Authors:** Emil Jędrzejewski, Miłosz Czuba, Adam Niemaszyk, Kamila Płoszczyca, Katarzyna Kaczmarczyk, Józef Langfort, Robert Gajda

**Affiliations:** 1 Warsaw Southern Hospital, 02-781 Warsaw, Poland; 2 Faculty of Rehabilitation, Józef Piłsudski University of Physical Education in Warsaw, 00-968 Warsaw, Poland; adam.niemaszyk@awf.edu.pl (A.N.);; 3 Department of Sports Theory, The Jerzy Kukuczka Academy of Physical Education, 40-065 Katowice, Poland; 4 Department of Kinesiology and Health Prevention, Jan Dlugosz University, 42-217 Czestochowa, Poland; gajda@gajdamed.pl

**Keywords:** hypercholesterolemia, hypoxia, intermittent hypoxic training, obesity

## Abstract

Obesity and overweight conditions, frequently accompanied by hypercholesterolemia, are major risk factors for cardiovascular disease. Lifestyle interventions remain the cornerstone of non-pharmacological treatment; however, their effectiveness in improving lipid profiles is limited. Intermittent hypoxic training (IHT) has recently emerged as a potential strategy to enhance metabolic outcomes. This study aimed to evaluate the effects of a 4-week intensive IHT program combined with a calorie-restricted diet on lipid profile and body composition in men with overweight or obesity and secondary hypercholesterolemia. Twenty physically inactive men (35.3 ± 5.4 years) were randomly assigned to either a hypoxic group (H, *n* = 10) or a normoxic control group (C, *n* = 10). Both groups followed the same training protocol and diet, differing only in environmental training conditions. Body composition, resting metabolic rate, and blood lipid parameters (total cholesterol, TC; high-density lipoprotein cholesterol, HDL-C; low-density lipoprotein cholesterol, LDL-C; non-high-density lipoprotein cholesterol, non-HDL-C; Triglycerides, TG) were assessed before and after the intervention. Compared with the C group, participants in the H group achieved significantly greater reductions in body mass (−5.4% vs. −2.6%, *p* < 0.05) and fat mass (−14.7% vs. −7%, *p* < 0.01). IHT also induced marked decreases in TC (−22.6%, *p* < 0.001), LDL-C (−25.8%, *p* < 0.001), non-HDL-C (−26.5%, *p* < 0.001), and TG (−31.4%, *p* < 0.01), along with a significant improvement in the atherogenic index of plasma (AIP, −24.4%, *p* < 0.05). In contrast, the C group showed only non-significant downward trends. No significant changes in HDL-C were observed in either group. These findings suggest that IHT combined with dietary restriction produces more favorable changes in lipid profile and body composition than normoxic training. IHT may therefore represent a promising adjunct to conventional lifestyle-based interventions in the management of obesity-related hypercholesterolemia.

## 1. Introduction

Obesity, characterized by excessive accumulation of adipose tissue and a chronic inflammatory state in the body, is one of the most pressing health challenges of the 21st century. Clinically, obesity is defined as a body mass index (BMI) ≥ 30 kg/m^2^, while overweight refers to BMI values between 25 and 29.9 kg/m^2^ (World Health Organization). Although the causes of obesity are complex, involving genetic, environmental, lifestyle, socioeconomic, as well as other factors, it most commonly develops in connection with a long-term positive energy balance and/or insufficient physical activity [1].

Obesity is associated with numerous metabolic disturbances, including secondary hypercholesterolemia, which poses a significant burden on public health by substantially contributing to cardiovascular morbidity and mortality worldwide [2]. Obesity-related hypercholesterolemia is characterized by elevated levels of low-density lipoprotein cholesterol (LDL-C), total cholesterol (TC), and triglycerides (TG), accompanied by reduced levels of high-density lipoprotein cholesterol (HDL-C). This dyslipidemic profile increases the risk of cardiovascular disease (CVD) [3,4].

The cornerstone of non-pharmacological treatment of obesity and hypercholesterolemia continues to involve lifestyle interventions, such as calorie-restricted diets and regular physical activity. Nevertheless, the lipid profile improvements achieved through these methods in obese individuals are often limited and depend on the extent of weight loss [5,6]. As a result, there is growing interest in complementary strategies that could enhance the effectiveness of conventional non-pharmacological approaches, such as diet and regular physical exercise.

A novel approach aims to support classical treatments by using a hypoxic environment as a therapeutic tool to aid in the treatment and prevention of obesity and to improve blood lipid profiles. One such strategy is intermittent hypoxic training (IHT)—a method that combines physical exercise and controlled exposure to air with reduced oxygen content (typically FiO_2_ 13–16%), simulating moderate-altitude conditions [7]. Unlike intermittent hypoxic exposure (IHE)—which exposes individuals to a reduced oxygen environment without engaging them in physical exercise—IHT integrates muscular effort with systemic hypoxia, which may lead to deeper metabolic changes, including lipid metabolism, endothelial function, and mitochondrial activity [8,9].

As a result, IHT may exert beneficial effects on parameters such as fat mass and lipid profile, particularly in individuals with obesity [10]. These health-promoting outcomes are thought to stem from the influence of hypoxia on leptin levels, adipocytokine secretion, metabolic rate, fat oxidation, and mitochondrial function [11,12]. Evidence also suggests that repeated hypoxic exposure activates the expression of hypoxia-inducible factor (HIF-1), further supporting the therapeutic potential of IHT in obesity management [1,13].

However, previous studies investigating the effectiveness of IHT in regulating lipid metabolism in overweight and obese individuals have reported conflicting results. A recent meta-analysis by He et al. [10] found that, compared with normoxic training, IHT more effectively reduced fat mass and improved BMI in middle-aged and older adults. Similarly, Ramos-Campo et al. [14] observed beneficial effects of hypoxia on body weight, fat mass, waist-to-hip ratio (WHR), waist circumference, and lipid profile, with stronger effects under hypoxia than normoxia for triglyceride reduction and muscle mass gain. Haufe et al. [15] confirmed a reduction in fat mass and improvements in insulin sensitivity markers, but without significant changes in cholesterol levels. In contrast, Costalat et al. [16] demonstrated improvements in total cholesterol and HDL/LDL fractions after only 10 days of passive hypoxic exposure. Additional evidence from animal studies further supports these findings: Wang et al. [17] reported positive changes in body weight, glucose tolerance, and lipid profile after 4 weeks of hypoxic training.

However, not all studies confirm the beneficial effects of IHT. In our own previous research [18], the most favorable changes in lipid profile were observed in participants exposed to prolonged passive hypoxia, rather than in those undergoing hypoxic training with a controlled diet. Similarly, Ghaith et al. [19] reported no improvements in body composition or lipid profile after 8 weeks of IHT, most likely due to the absence of dietary control and negative energy balance. Other investigations without dietary control [20,21] likewise failed to confirm the effectiveness of IHT in improving lipid profile or reducing fat mass.

It is important to note that, unfortunately, IHT protocols vary significantly in terms of the severity of hypoxia, duration, and number of cycles, all of which influence physiological outcomes and complicate comparisons across studies. Moreover, most trials assessing the effects of IHT on lipid profile and body composition in overweight and obese individuals have not incorporated a controlled hypocaloric diet—despite dietary intervention being a cornerstone in the management of obesity and hypercholesterolemia. Even in our previous study [18], where diet was controlled, the absence of a negative energy balance may explain the lack of favorable changes in lipid parameters.

Therefore, the aim of this study was to examine changes in blood lipid profile markers and body composition in obese men with secondary hypercholesterolemia resulting from an unhealthy lifestyle, without comorbidities, in response to a 4-week high-intensity training program conducted under normobaric hypoxia. We hypothesized that intensive training under normobaric hypoxic conditions (IHT), combined with a controlled diet with a negative energy balance, would lead to beneficial changes in the lipid profile, atherogenic indices, and body composition. Furthermore, we expected that the effects of training under hypoxia would be more pronounced than those achieved under normoxia.

## 2. Results

### 2.1. Changes in Body Mass and Body Composition

**Figure 1:**
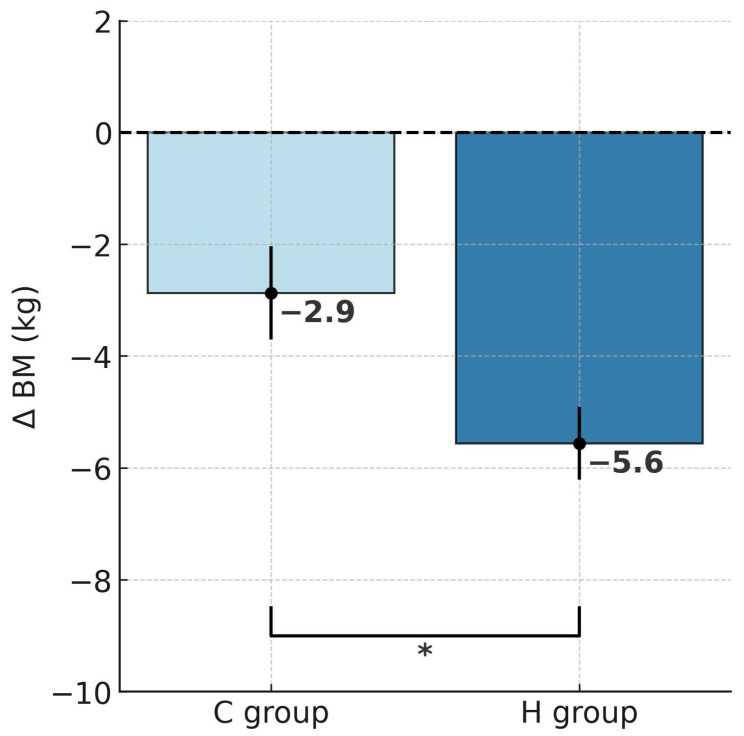
Post-intervention changes in body mass in hypoxic and control groups; * *p* < 0.05, significant differences between groups. Abbreviations: ΔBM—change in body mass, H group—hypoxic training group, C group—control group.

**Figure 2:**
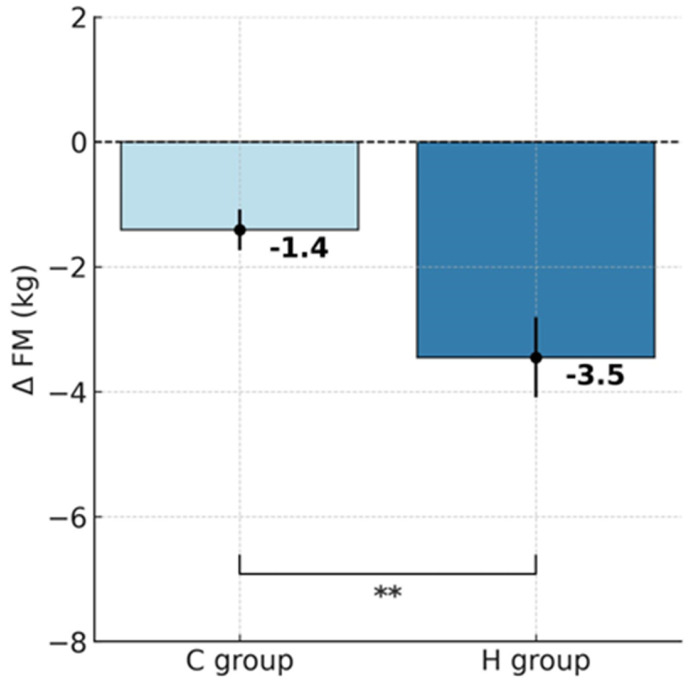
Post-intervention changes in fat mass in hypoxic and control groups; ** *p* < 0.01, significant differences between groups. Abbreviations: ΔFM—change in fat mass, H group—hypoxic training group, C group—control group.

**Figure 3:**
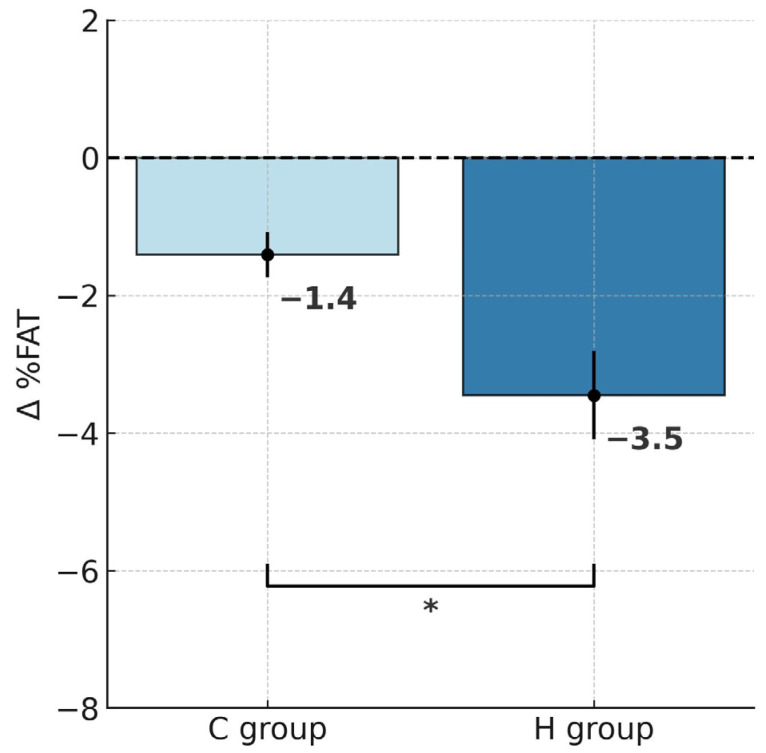
Post-intervention changes in percentage of body fat in hypoxic and control groups; * *p <* 0.05, significant differences between groups. Abbreviations: Δ%FAT—change in percentage of body fat, H group—hypoxic training group, C group—control group.

ANOVA with repeated measures for group × training interactions showed statistically significant differences in body mass (BM; F = 6.452, *p* < 0.05), body fat percentage (%FAT; F = 8.035, *p* < 0.01) and fat mass (FM; F = 9.461, *p* < 0.01). According to Tukey’s post hoc test, the H group showed a significant reduction in BM by 5.4% (*p* < 0.001), whereas the C group also experienced a significant decrease, though notably smaller, by 2.6% (*p* < 0.01; Table 1). Analysis of delta BM values using the t-test demonstrated a significantly greater BM reduction in the H group that trained under hypoxic conditions compared to the C group (t = 2.539, *p* < 0.05; Figure 1). Similar patterns were also observed for FM. After the training, FM decreased significantly by 14.7% in the H group (*p* < 0.001) and by 7% in the C group (*p* < 0.01). Moreover, the between-group comparison showed a significantly greater FM reduction in the H group (t = 3.075, *p* < 0.01; Figure 2). In terms of %FAT, a significant reduction of 10.3% was observed only in the H group (*p* < 0.001). The C group showed a downward trend that did not reach statistical significance (*p* = 0.06). However, the t-test indicated that the reduction in %FAT was significantly greater in the H group compared to the C group (t = 2.834, *p* < 0.05; Figure 3).

### 2.2. Changes in Lipid Profile

**Figure 4:**
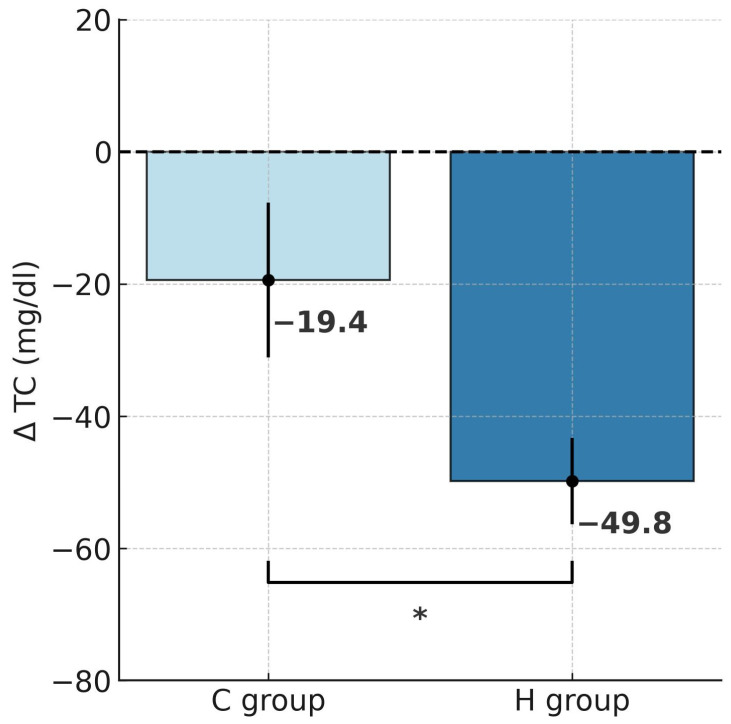
Post-intervention changes in total cholesterol concentrations in hypoxic and control groups; * *p* < 0.05, significant differences between groups. Abbreviations: ΔTC—change in total cholesterol concentrations, H group—hypoxic training group, C group—control group.

**Figure 5:**
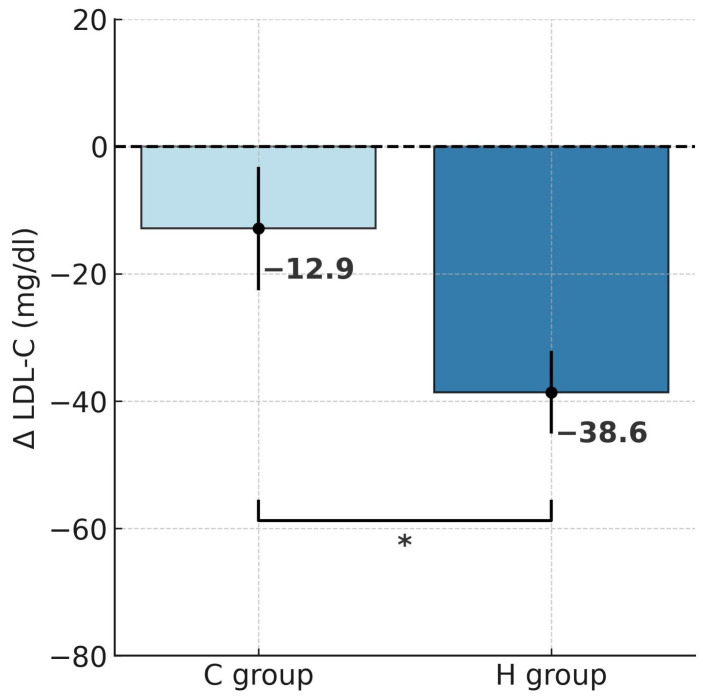
Post-intervention changes in LDL cholesterol concentrations in hypoxic and control groups; * *p* < 0.05, significant differences between groups. Abbreviations: ΔLDL-C—change in low-density lipoprotein cholesterol, H group—hypoxic training group, C group—control group.

**Figure 6:**
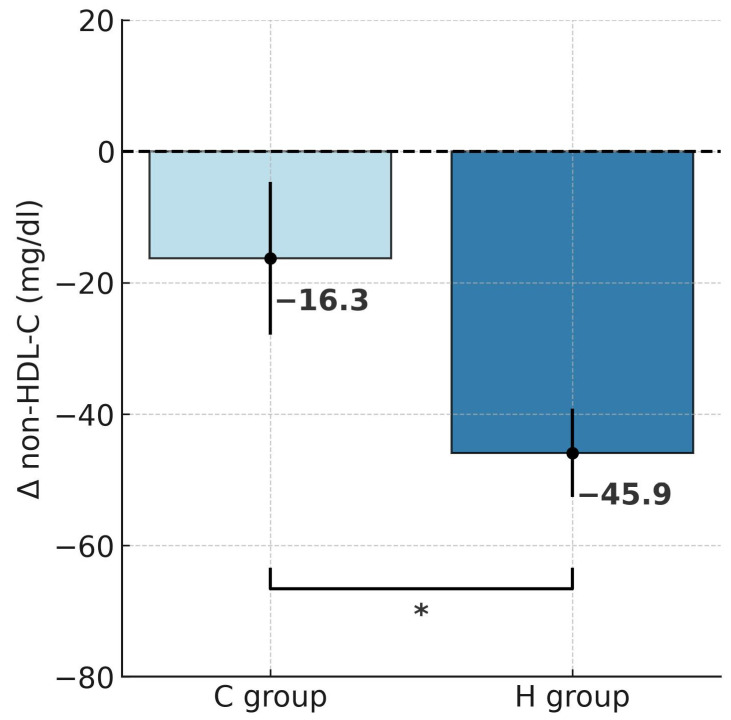
Post-intervention changes in non-HDL cholesterol concentrations in hypoxic and control groups; * *p* < 0.05, significant differences between groups. Abbreviations: Δnon-HDL-C—change in non-high-density lipoprotein cholesterol, H group—hypoxic training group, C group—control group.

**Figure 7:**
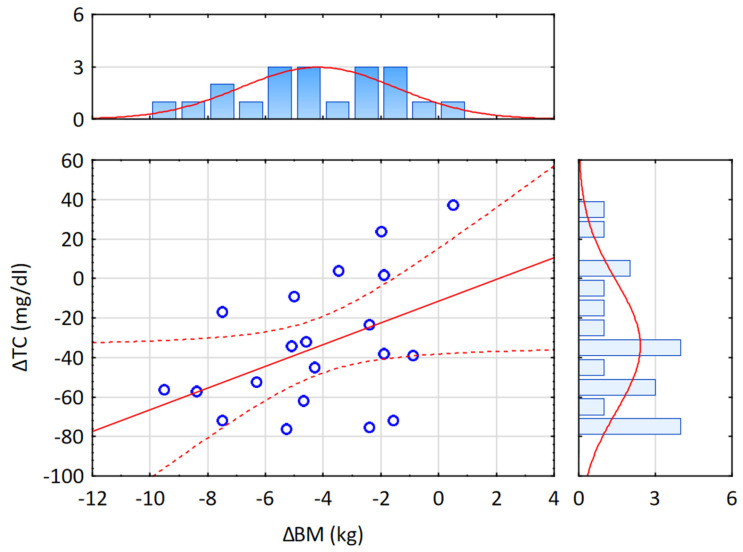
Correlation between delta values for body mass (ΔBM) and delta values of total cholesterol (∆TC) after the experiment in both groups. Abbreviations: ΔBM—change in body mass, ΔTC—change in total cholesterol; solid line—regression line described by the equation: ΔBM= −2.962 + 0.03621 × ΔTC; dot line—95% confidence interval; circle—individual data points for each participant.

Repeated-measures ANOVA revealed a significant effect of hypoxic training on TC (F = 5.134, *p* < 0.05), LDL-C (F = 4.850, *p* < 0.05), non-HDL-C (F = 4.843, *p* < 0.05), and TG (F = 17.29, *p* < 0.001). Tukey’s post hoc analysis indicated a significant reduction in TC by 22.6% following training in hypoxia (*p* < 0.001; Table 2). The t-test further confirmed that this decrease was significantly greater in the H group compared to the C group (t = 2.267, *p* < 0.05; Figure 4). HDL-C did not change significantly in either group. Notably, LDL-C decreased markedly by 25.8% in the H group (*p* < 0.001), and the reduction was significantly larger under hypoxia compared with normoxia (t = 2.202, *p* < 0.05; Figure 5). Similarly, non-HDL-C levels fell by 26.5% in the H group (*p* < 0.001), with between-group comparison showing a significantly greater reduction in the hypoxic condition (t = 2.201, *p* < 0.05; Figure 6). Because TG concentrations in the control group were not normally distributed, the Wilcoxon test was applied for paired data (before vs. after intervention within a group), and the Mann–Whitney U test was used to compare independent samples (between groups H and C). Results showed a significant 31.4% reduction in TG in the H group (Z = 2.599, *p* < 0.01; Median (IQR25-75) = 146.5 (101.0–179.1) vs. 98.0 (62.0–116.0), while no significant changes were observed in the C group (Median (IQR25-75) = 152.9 (129.4–434.0) vs. 158.0 (98.5–256.0)) after the intervention. The magnitude of TG reduction did not differ significantly between groups (U = 28.5, *p* = 0.112). Correlation analysis demonstrated a significant association between ∆BM and ∆TC (r = 0.45, *p* < 0.05; Figure 7). Although a downward trend in atherogenic indices (AIP, CRI-I, and CRI-II) was observed in both groups, the Wilcoxon test confirmed a significant 24.4% reduction in AIP in the H group (Z = 2.394, *p* < 0.05; Table 3).

## 3. Discussion

To the best of our knowledge, this is the first study to evaluate the effects of intermittent hypoxic training (IHT) combined with a controlled hypocaloric diet on lipid profile and body composition in individuals with secondary hypercholesterolemia.

Our results demonstrated that 4 weeks of training under hypoxic conditions, together with a controlled reduction diet, led to significantly greater decreases in BM (−5.4% vs. −2.6%) and FM (−14.7% vs. −7%) compared with training under normoxia. A significant reduction in %FAT (−10.3%) was observed only in the hypoxia group (H). Following hypoxic training, marked decreases were also noted in TC (−22.6%), LDL-C (−25.8%), and non-HDL-C (−26.5%), which were approximately twice as great as those observed in the normoxic group (C). In addition, the H group showed a significant reduction in TG levels, which also resulted in a significant improvement in the atherogenic index of plasma (AIP). In contrast, the C group displayed only downward trends in these parameters, with no statistically significant changes. A significant correlation was also identified between ∆BM and ∆TC (r = 0.45).

It is well established that regular physical activity is one of the most important factors influencing improvements in blood lipid profile [22] and the reduction of excessive body fat [23]. However, these beneficial effects depend on the type, frequency, intensity, and duration of exercise [24,25]. It has also been demonstrated that lipid profile may be modified by diet and supplementation [26,27,28]. In addition, environmental conditions affect changes in lipid profile and cardiovascular disease (CVD) risk [29].

Populations living at high altitudes have been shown to have a lower risk of CVD and significantly reduced mortality, which has been linked to lipid profile characteristics [30]. Several studies have reported that native high-altitude residents exhibit lower TC and LDL-C levels and higher HDL-C levels [31,32,33]. Improvements in lipid profile have also been observed among participants of high-altitude expeditions [34,35], indicating that even short stays at high elevations (above 4000 m) may induce favorable changes. Similar effects have been noted at moderate altitudes [36,37]. Continuous exposure (days to weeks) at these altitudes has been associated with significant reductions in TC concentrations [37,38]. Most studies found a downward trend in LDL-C, while Greie et al. [37,38] reported a significant reduction in LDL-C (10–14%) after only several days of exposure to moderate altitude. These changes were accompanied by significant decreases in TC (6–13%) [37,38]. Findings regarding HDL-C were inconsistent, with some studies reporting increases and others no change [37]. Importantly, these studies were conducted in physically inactive individuals.

Interestingly, similar changes were also demonstrated in one of our previous studies in a group of athletes [18]. In this case, the effectiveness of the hypoxic stimulus depended strongly on the training method. Beneficial changes were observed in the group exposed to several hours per day at 2000 m for 3 weeks (live high—train low, LH–TL), whereas no changes were noted in the group performing IHT (two sessions per week at a simulated altitude of 3000 m). The lack of response in lipid profile after IHT may have been due to the participants being healthy men with normal lipid profiles, as well as the relatively low IHT dose.

Considering the results of the present study and previous findings on IHT, while emphasizing that a negative energy balance remains the cornerstone of non-pharmacological treatment for obesity and hypercholesterolemia [39], it must be concluded that the effectiveness of IHT can be reliably demonstrated only when combined with a controlled hypocaloric diet. Individuals with overweight or obesity generally maintain a positive energy balance, which means that physical activity alone—even when combined with hypoxia—without simultaneous modification of unhealthy eating habits, is insufficient to achieve significant metabolic effects. Moreover, physical activity may increase appetite, and without dietary control this may lead to greater energy intake [40,41]. This likely explains the lack of beneficial effects of IHT interventions on lipid profile reported in some studies.

Consequently, it should be acknowledged that the absence of a controlled diet ensuring a negative energy balance is a key factor limiting the potential for favorable changes in lipid profile in response to IHT interventions among individuals with overweight or obesity. This conclusion is supported by previous findings. In the study by Fernández Menéndez et al. [20], the lack of a controlled diet resulted in no significant changes in TC, BM, or FM following IHT. A similar outcome was reported by Klug et al. [21], where diet was also not monitored. The authors observed significant changes in LDL-C in both the IHT and control groups, along with a small reduction in body mass (~2 kg after 12 weeks). However, the magnitude of this reduction was markedly smaller compared to the results of the present study and was most likely due to the presence of a modest, unmonitored caloric deficit in the IHT group. The most advanced work in this area appears to be that of Kong et al. [42], who studied a group of obese women without introducing a controlled hypocaloric diet. Instead, dietary intake was only monitored, with participants instructed to maintain constant macronutrient proportions (60% carbohydrates, 35% fat, 15% protein), but without standardized meals. The absence of significant changes in BM and FM clearly indicates the lack of a caloric deficit during the intervention, which was reflected in the absence of improvements in lipid profile.

Our hypothesis regarding the crucial role of a negative energy balance in the non-pharmacological treatment of hypercholesterolemia in obesity is supported by the findings of this study, which demonstrated a significant correlation between ∆BM and ∆TC (r = 0.45; *p* < 0.05). This result indicates that the reduction in TC levels among overweight or obese individuals is closely dependent on body mass reduction. These findings are consistent with previous reports. Van Gaal et al. [5] showed that a 5–10% decrease in body mass leads to a reduction in LDL-C by approximately 15%, TG by 20–30%, and an increase in HDL-C by 8–10%. These results are comparable to the outcomes observed in the H group, where IHT combined with a hypocaloric diet produced a significant 14.7% reduction in BM, along with marked decreases in LDL-C (−26.5%) and TG (−31.4%), although no significant changes were found in HDL-C. Similarly, a meta-analysis by Poobalan et al. [6] indicated that in overweight or obese individuals, each 10 kg reduction in body mass may correspond to a decrease in TC of approximately 0.23 mmol/L (~9 mg/dL). Interestingly, in the H group subjected to IHT, we obtained considerably more favorable results (≈6 kg reduction in BM; ≈50 mg/dL reduction in TC), suggesting that hypoxia may accelerate cholesterol utilization and/or inhibit its synthesis.

From a molecular mechanism perspective, IHT activates hypoxia-inducible factor 1α (HIF-1α), which regulates the expression of genes responsible for cholesterol transport, fatty acid oxidation, and endothelial function [43]. It has also been suggested that the beneficial effect of IHT on blood lipid levels is related to an increased rate of lipid oxidation due to elevated expression of mRNA encoding the protein PGC-1α (peroxisome proliferator-activated receptor-gamma coactivator 1α), which induces mitochondrial biogenesis and plays a key role in regulating fatty acid oxidation in muscle [15,44]. Such adaptive changes are particularly important in the context of obesity, where lipid metabolism is often impaired due to insulin resistance, chronic inflammation, and hepatic steatosis [4,45].

The observed reduction in LDL-C following IHT can be explained by the simultaneous action of several mechanisms: activation of hypoxia-inducible factor 1α (HIF-1α) and increased expression of PGC-1α [46,47], which may promote enhanced fatty acid oxidation and greater utilization of lipids as an energy source [48]; upregulation of LDL receptor expression in hepatocytes, which accelerate lipoprotein catabolism and cholesterol clearance from the blood [49]. In addition, reductions in BM and FM associated with a negative energy balance decrease the release of free fatty acids and the hepatic synthesis of VLDL, thereby indirectly reducing circulating LDL-C [50]. These findings suggest that the effect of IHT is not solely a consequence of hypoxia, but rather the interaction between the hypoxic stimulus, physical exercise, and controlled diet. Moreover, these mechanisms may explain why LDL-C reductions in the H group were more than twice as great as those observed in the C group.

The significant reduction in blood TG levels observed after IHT in the present study is consistent with earlier findings from interventions conducted at moderate altitudes (2000–3000 m) [15,51]. This effect has been attributed to enhanced fat oxidation resulting from increased expression of PGC-1α, a protein responsible for mitochondrial biogenesis and fatty acid metabolism [15,44,48,52]. Haufe et al. [15] further suggested that the combination of hypoxia and physical exercise may exert a stronger effect on TG concentrations than either factor alone. It is worth noting that, in the present study, the intervention applied in the C group (training + diet) did not result in a significant reduction in blood TG levels.

In the present study, no significant changes in HDL-C concentrations were observed in either group. Rather, only a slight downward trend was noted, which contrasts with the findings of many earlier studies. The literature emphasizes that both adaptation to physical exercise and prolonged exposure to hypoxia may lead to an increase in HDL-C levels [18,53]. However, the body’s response to short-term hypoxic exposure and intermittent hypoxic training (IHT) remains inconclusive [18,54]. The lack of change in HDL-C observed in our study was most likely due to the substantial caloric deficit, which induced rapid body mass reduction. Such conditions prevent metabolic stabilization and do not favor increases in HDL-C [18,55]. In the classic study by Thompson et al. [56], women following a hypocaloric diet exhibited a decline in HDL-C levels proportional to the degree of negative energy balance. Regression analysis revealed that greater relative BM loss and faster FM reduction were associated with a more pronounced decrease in HDL-C. Consequently, restricted caloric intake may not only inhibit increases in HDL but even lower its circulating levels [56].

Furthermore, the absence of an increase in HDL-C in our study, as well as in the study by Gao et al. [55] conducted in women with obesity and metabolic syndrome, may also be attributable to the restriction of dietary fat intake to 20%. This assumption is supported by earlier findings of Brinton et al. [57], who demonstrated that switching from a high-fat to a low-fat diet (42% vs. 9% fat) lowers HDL-C levels by reducing the transport rate of HDL apolipoproteins. In our study, dietary fat intake was reduced to 20% in favor of increasing protein intake to 30% as part of the hypocaloric diet. This strategy was intended to provide an anticatabolic effect [58], which was confirmed by the absence of significant changes in FFM and RMR in either group.

### Limitations

This study has several limitations that should be considered when interpreting the results. First, the sample size was relatively small (*n* = 20), which limits the generalizability of the findings. However, it should be emphasized that despite the small number of participants, strict dietary control was implemented—participants received a controlled isocaloric diet throughout the study—which increased the reliability of the data and minimized potential confounding factors. Second, the intervention period (4 weeks) was relatively short and did not allow for an assessment of the long-term effectiveness of the observed changes. However, this study is part of a broader project aimed at developing and implementing a training protocol designed to effectively improve lipid profile, body composition and cardiovascular variables in individuals with obesity-related hypercholesterolemia. Since the protocol requires participants to be accommodated in a controlled environment (due to hypoxic training conditions and dietary supervision), it was intentionally designed to be of relatively short duration to ensure its feasibility and potential applicability in real-world practice. Future studies should include longer interventions and follow-up assessments to verify whether the favorable metabolic adaptations induced by hypoxic training persist over time. We suppose that the durability of these effects will largely depend on whether participants adopt lasting changes in dietary habits and lifestyle, which are essential for maintaining body mass reduction and improving lipid profile.

Our results demonstrate that the proposed protocol alone, without pharmacotherapy, can produce significant benefits in physically inactive men with overweight or obesity and secondary hypercholesterolemia, in the absence of other medical conditions. Future studies should also investigate the potential synergistic effects of combining IHT with pharmacological treatment, particularly in patients with more advanced obesity or metabolic disorders.

Another limitation is the restriction of the study population to men aged 20–40 years. Women were intentionally excluded to avoid the influence of hormonal fluctuations related to the menstrual cycle on lipid metabolism outcomes. While this decision increased the internal validity of the study, it also limited the applicability of the results to women and older individuals. In addition, no control group was included that followed only a hypocaloric diet, which makes it difficult to fully separate the effects of diet alone from those resulting from the combination of exercise and energy deficit. However, our primary objective was to compare the combined effects of identical training and nutritional programs conducted under normobaric hypoxic and normoxic conditions. By maintaining the same dietary and exercise protocols across both groups, we were able to isolate the specific impact of the environmental factor—hypoxia—on the lipid profile and body composition. It should further be noted that although dietary restriction is the foundation of intervention effectiveness, its impact alone would not be expected to exceed the results obtained in the C group (exercise under normoxic conditions + diet).

The present study did not include molecular analyses that could clarify the specific pathways mediating the lipid-modulating effects of hypoxic exercise combined with caloric restriction. Further investigations are warranted to explore these mechanisms, including gene expression, enzymatic activity, and signaling pathways involved in lipid metabolism under hypoxic conditions.

## 4. Materials and Methods

### 4.1. The Participants

The study involved 20 physically inactive men aged 20–40 years with overweight or obesity and secondary hypercholesterolemia resulting from an unhealthy lifestyle (i.e., physical inactivity and poor dietary habits), without coexisting medical conditions. Participants were randomly assigned to either an experimental group (H group) or a control group (C group) using an Excel-based number generator. The randomization list was prepared by an independent researcher who was not involved in participant recruitment or assessment to ensure allocation concealment. The inclusion criteria for both groups were: (1) clinically confirmed hypercholesterolemia; (2) age 20–40 years; (3) absence of chronic diseases; (4) resting systolic blood pressure <160 mmHg and diastolic blood pressure <100 mmHg. The exclusion criteria were: (1) use of illicit drugs, alcohol consumption, or smoking; (2) stage 2 or higher hypertension; (3) premature termination of the exercise test or training program. The baseline characteristics of the participants are presented in Table 4.

All participants underwent a comprehensive medical examination to confirm the absence of contraindications to exercise under hypoxic conditions. Written informed consent was obtained from all participants prior to enrollment, in accordance with ethical guidelines. The study adhered to the principles of the Declaration of Helsinki and was approved by the Bioethics Committee at the University of Zielona Góra, Poland (Resolution No. 6/2023).

### 4.2. Study Design

The experimental protocol consisted of two measurement series: baseline testing (S1) and post-intervention testing (S2). The required sample size was determined using an a priori power analysis performed with G*Power 3.1 software. Assuming an acceptable statistical power of 1 − β = 0.80, a significance level of α = 0.05, and an effect size of 0.30, the analysis indicated that a total of *n* = 20 participants would be required to test each research hypothesis. To minimize hormonal variability and its potential impact on lipid profile markers, only male participants were recruited, thereby ruling out any confounding influence of the menstrual cycle on lipid-related variables [59].

Both groups followed the same training program and received an identical isocaloric diet, designed and supervised by a certified clinical dietitian. The only difference between groups was the environmental condition in which the training was conducted. Group H performed the training under normobaric hypoxia (simulated altitude conditions), while Group C trained under normoxic conditions. The study design did not include a diet-only control group, as the primary objective was to assess the combined effect of exercise and diet performed under different environmental conditions (normobaric hypoxia vs. normoxia). All training sessions were supervised, and adherence to both the exercise and dietary protocols was monitored throughout the 4-week intervention period.

### 4.3. Testing Protocol

In both series of testing (S1, S2), body mass and body composition were taken between 8:00 and 8:30 a.m., after an overnight fast. Body height was measured using an anthropometer with an accuracy of 0.5 cm, and body composition was estimated using bioelectrical impedance analysis (InBody 220, Biospace, Seoul, South Korea). To ensure consistency, participants were instructed to: (1) fast for at least 8 h, (2) consume a minimum of 2 L of water the day before, (3) avoid physical exertion for at least 8 h, (4) refrain from coffee or alcohol intake for at least 12 h, and (5) abstain from diuretics for 24 h. Participants were also asked to empty their bladder immediately before measurement [60]. The system provided estimates of fat mass (FM), body fat percentage (%FAT), and fat-free mass (FFM).

Next, resting metabolic rate (RMR) was measured using indirect calorimetry with a face mask (Cortex Metalyser 3B, Leipzig, Germany), a device shown to be reliable and valid in previous studies [61]. The procedure followed a standard protocol. The gas analyzer was calibrated before each session with ambient air and standard gas (5% CO_2_, 15% O_2_), and flow calibration was performed using a 3 L syringe (Hans Rudolph, Shawnee, KS, USA). Participants fasted for 12 h and avoided physical activity before testing. They rested awake in a supine position in a quiet and temperature (22–23 °C)- and humidity-controlled room. Energy expenditure was measured in all participants over a 15 min period, excluding the first 5 min allocated for preparation and stabilization. Measurements that did not achieve at least 5 min of steady state (with VO_2_ and VCO_2_ variability not exceeding ±10%) were excluded from the analysis. In such cases, the measurement was repeated the following day. At all times during the measurement, gas volume variability was required to remain at or below 10% at least for 5 min [62].

The venous blood sample was obtained between 8:30 and 9:00 a.m., immediately after the RMR assessment. The blood sample (10 mL) was drawn from the cubital vein to determine total cholesterol (TC), high-density lipoprotein cholesterol (HDL-C), and triglycerides (TG) (Cobas 6000/c501, Roche, Mannheim, Germany). The low-density lipoprotein cholesterol (LDL-C) was calculated using the Friedewald formula: LDL-C = TC − (TG/5 + HDL-C) [63]. Additionally, the atherogenic index (AIP) and lipid ratios (CRI-I, CRI-II) were calculated using the following formulas [64,65]:

- Atherogenic index of plasma (AIP) = log10 (TG/HDL-C);
- Castelli’s risk index I (CRI-I) = TC/HDL-C;
- Castelli’s risk index II (CRI-II) = LDL-C/HDL-C.

After blood sampling, all participants consumed a standardized light meal (350 kcal; 15 g protein, 12.3 g fat, 44.2 g carbohydrates). Two hours later, they performed a graded exercise test on a cycle ergometer (Excalibur Sport, Lode BV, Groningen, The Netherlands), individually adjusted for each participant. The test started at 50 W and increased by 25 W every 3 min until volitional exhaustion. During the final 15 s of each workload stage, capillary blood samples were collected from the fingertip to measure blood lactate concentration (LA; LABTREND, BST Bio Sensor Technology GmbH, Berlin, Germany). Lactate data were analyzed to assess the kinetics of blood LA concentration and to determine the individual lactate threshold using the Dmax method [66]. The lactate threshold was then used to prescribe individualized training workloads for the intervention sessions.

### 4.4. Training Program

The training program included 4 weeks with progressively increasing training loads. Both groups followed the same training routines, with individually adjusted intensity zones. Training was carried out three times a week, on Mondays, Wednesdays, and Fridays. Each training session in the laboratory included a 5 min warm-up, 80 min main part and 5 min cool-down. Intensity during these sessions was tailored to each study participant based on the heart rate at lactate threshold (HRLT). The warm-up during all training sessions was performed at 70–80% HRLT, while the main part was set at 90–100% HRLT. In the main part of each session, the participants performed four 10 min repetitions on a cycling ergometer, with a 10 min break between each. During the break, they performed exercises targeting the shoulder girdle, abdominal muscles, and back muscles, to maintain high intensity throughout the session. The cool-down included 5 min of continuous exercise at 65–70% HRLT. In all participants, exercise intensity was monitored by recording heart rate using a heart rate monitor (Polar Pacer, Polar Electro Oy, Kempele, Finland). Additionally, in the H group (training under hypoxic conditions), SpO_2_ was also monitored (3150 WristOx2, Nonin Medical Inc., Plymouth, MN, USA). All training sessions were conducted by a personal trainer and supervised by the research team.

### 4.5. Diets During the Experiment

Throughout the experiment, all participants followed the same training schedule and diet. During the experiments, the participants consumed a controlled mixed diet (45.6 ± 4.2% CHO, 23.4 ± 2.9% Fat, 30.6 ± 2.9 % Pro; Table 5). The daily caloric intake was maintained at around 2.515 ± 40.6 kcal. This caloric intake was individually adjusted based on measurements of resting metabolic rate obtained using indirect calorimetry (Table 1). The calories were divided into five meals: breakfast, second breakfast, lunch, afternoon snack and dinner. Dietary adherence was strictly controlled throughout the 4-week intervention period. All meals were prepared and portioned individually. Participants were accommodated on-site for the entire duration of the intervention, which allowed for direct supervision of meal consumption and full control over dietary intake. Participants were required to drink at least 2.5 L of water on non-training days and 3.5 L on training days [67]. No alcohol, dietary supplements, or medications were used by the participants.

### 4.6. Statistical Analysis

All data were analyzed using Statistica v.13 (StatSoft). Results are presented as arithmetic means (x) ± standard deviations (SD). The normality of distributions was verified using the Shapiro-Wilk test, and homogeneity of variance was assessed with Levene’s test. When assumptions for parametric testing were met, a two-way repeated-measures ANOVA was applied. If significant effects were observed, post hoc analyses were conducted. To compare between-group differences in delta values of the studied variables, independent-samples t-tests were used. When normality assumptions were violated, the Wilcoxon test was applied as a non-parametric alternative for paired data (before vs. after intervention within a group), and the Mann–Whitney U test was used to compare independent samples (between groups H and C). Pearson’s correlation analysis was performed to assess relationships between selected variables. For all analyses, statistical significance was set at *p* < 0.05.

## 5. Conclusions

This study found that training under hypoxic conditions, combined with a controlled hypocaloric diet, results in significantly greater reductions in body mass and fat mass compared with training under normoxia. A significantly greater decrease in TC, LDL-C, non-HDL-C, and triglyceride concentrations was also observed in the hypoxia group compared to the control group. In addition, the atherogenic index of plasma (AIP) improved only in the group subjected to IHT, further confirming the beneficial effect of IHT on lipid profile. Despite clear improvements in most lipid fractions, no significant changes in HDL-C levels were observed, highlighting the need for further research in this area. Importantly, the results confirm that a negative energy balance is essential for effective improvements in lipid profile and body mass reduction in individuals with obesity and secondary hypercholesterolemia. Without dietary control, training interventions—including those conducted under hypoxia—will not yield the expected metabolic benefits. Overall, the findings suggest that IHT may serve as a promising adjunct to non-pharmacological therapy; however, lasting lifestyle modification remains crucial for maintaining the achieved effects.

## Figures and Tables

**Table 1:** Body mass and composition of participants before (S1) and after training (S2) in the hypoxic training (H) and control (C) groups.

Variables	H Group		C Group	
	Before (S1)	After (S2)	Before (S1)	After (S2)
BM (kg)	103.1 ± 11.8	97.5 ± 11.3 ***	99.5 ± 7.8	96.7 ± 7.8 **
%FAT	33.6 ± 7.7	30.2 ± 8.1 ***	32.9 ± 4.9	31.5 ± 4.1 #
FM (kg)	35.3 ± 10.7	30.1 ± 10.1 ***	32.8 ± 5.8	30.5 ± 4.8 **
FFM (kg)	67.8 ± 5.5	67.5 ± 6.1	66.7 ± 6.3	66.1 ± 6.2
RMR (kcal/day)	2395.0 ± 215.3	2411.5 ± 205.6	2337.9 ± 238.5	2310.8 ± 135.8

Abbreviations: BM—body mass; %FAT—fat content; FM—fat mass; FFM—fat-free mass; RMR—resting metabolic rate; # *p* < 0.06, ** *p* < 0.01, *** *p* < 0.001 before vs. after training.

**Table 2:** Lipid profile of participants before (S1) and after training (S2) in the hypoxic training (H) and control (C) groups.

Variables	H Group		C Group	
	Before (S1)	After (S2)	Before (S1)	After (S2)
TC (mg/dL)	220.2 ± 26.9	170.4 ±19.7 ***	230.4 ± 24.1	211.0 ± 31.1 ###
HDL-C (mg/dL)	46.9 ± 7.3	41.9 ±6.4	46.1 ±14.2	43.3 ± 10.3
LDL-C (mg/dL)	149.1 ± 22.3	110.5 ± 15.8 ***	141.9 ± 24.8	129.0 ± 16.5
non-HDL-C (mg/dL)	173.1 ± 26.7	127.2 ± 22.5 ***	183.9 ± 26.3	167.6 ± 33.7 #
TG (mg/dL)	139.8 ± 51.1	95.9 ± 34.2 **	236.5 ± 164.9	210.3 ± 150.9

Abbreviations: TC—total cholesterol; HDL-C—high-density lipoprotein cholesterol; LDL-C—low-density lipoprotein cholesterol; non-HDL-C non-high-density lipoprotein cholesterol; TG—triglycerides; ** *p* < 0.01, *** *p* < 0.001 before vs. after training; # *p* < 0.05, ### *p* < 0.001 significant differences after training (group H vs. group C).

**Table 3:** Atherogenic indices in participants before (S1) and after intervention (S2) in hypoxia (H) and control groups (C).

Variables	H Group		C Group	
	Before (S1)	After (S2)	Before (S1)	After (S2)
AIP	0.45 ± 0.18	0.34 ± 0.18 *	0.64 ± 0.35	0.61 ± 0.36
CRI-I	4.78 ± 0.94	4.1 ± 0.82	5.38 ± 1.57	5.17 ± 1.81
CRI-II	3.26 ± 0.82	2.7 ± 0.66	3.32 ± 1.07	3.13 ± 0.87

AIP—atherogenic index of plasma: log10(TG/HDL-C); CRI-I—Castelli’s risk index I: TC/HDL-C; CRI-II—Castelli’s risk index II: LDL-C/HDL-C; * *p* < 0.05 before vs. after training.

**Table 4:** Baseline characteristics of the participants.

Variables	H Group	C Group
Age (years)	37.3 ± 5.8	33.3 ± 4.4
Body height (cm)	178.7 ± 5.2	177.5 ± 5.3
Body mass (kg)	103.1 ± 11.8	99.5 ± 7.9
Body fat (%)	33.6 ± 7.7	32.9 ± 4.9
Systolic blood pressure (mmHg)	137.1 ± 6.1	135.0 ± 12.5
Diastolic blood pressure (mmHg)	81.1 ± 9.2	84.8 ± 12.7

**Table 5:** Nutritional composition during the study.

Protein (g)	Fat (g)	Carbohydrates (g)	Caloric Intake (kcal)
191.2 ± 16.4	65.6 ± 8.4	287.3 ± 26.8	2515.7 ± 40.6

## Data Availability

The original contributions presented in this study are included in the article. Further inquiries can be directed to the corresponding author(s).

## Abbreviations

%FAT	Body fat percentage
AIP	Atherogenic index of plasma
BH	Body height
BM	Body mass
BMI	Body mass index
CRI-I	Castelli Risk Index I
CRI-II	Castelli Risk Index II
CVD	Cardiovascular disease
FFM	Fat-free mass
FM	Fat mass
HDL-C	High-density lipoprotein cholesterol
HIF-1	Hypoxia-inducible factor
HRLT	Heart rate at lactate threshold
IHE	Intermittent hypoxic exposure
IHT	Intermittent hypoxic training
LDL-C	Low-density lipoprotein cholesterol
LH–TL	Live high—train low
PGC-1α	Peroxisome proliferator-activated receptor-gamma coactivator 1α
RMR	Resting metabolic rate
SD	Standard deviations
TC	Total cholesterol
TG	Triglycerides
WHO	World Health Organization
WHR	Waist-to-hip ratio
